# Cognitive and human factors in legal layperson decision making: Sources of bias in juror decision making

**DOI:** 10.1177/00258024221080655

**Published:** 2022-02-17

**Authors:** Lee J. Curley, James Munro, Itiel E. Dror

**Affiliations:** 1 Faculty of Arts and Social Sciences, School of Psychology and Counselling, 5488 the Open University, Milton Keynes, UK; 2 UCL JDI Centre for the Forensic Sciences, 4919 University College London, London, UK

**Keywords:** Pre-trial bias and attitudes, pre-trial publicity, cognitive bias, expert witness bias, juror decision making, jury decision making, deliberations

## Abstract

Juries in adversarial courts are tasked with several responsibilities. They are asked to: 1) assess the credibility and reliability of the evidence presented; 2) deliberate; 3) and then reach a decision. Jurors are expected to evaluate said evidence in a rational/impartial manner, thus allowing the defendant their right to a fair trial. However, psychological research has shown that jurors are not rational and can reach inaccurate decisions by being biased by certain factors. The aim of the current review was to explore the potential sources from which biases are introduced into the jury. Three main sources of bias were focussed upon: 1) pre-trial bias; 2) cognitive bias; 3) bias from external legal actors (expert witnesses). Legal scholars commonly cite deliberations as a method of attenuating individual juror bias, this claim is evaluated in the review. The review concludes that bias is a multifaceted phenomenon introduced from many different elements, and that several sources of bias may interact with one another during a jury trial to cause the effects of bias to snowball. Four recommendations are made: 1) juror selection should be utilised to create heterogenous juries that challenge problematic biases from individual jurors; 2) increase the quality of expert testimony through training; 3) procedures such as Linear Sequential Unmasking should be adopted by expert witnesses to filter out some sources of bias; 4) legal professionals and jurors should be educated about the effects that biases may have on decision making; 5) more research into bias in jurors is needed.

## Introduction

Jurors are legal laypersons who are expected to hear evidence, and then evaluate its credibility and reliability, to reach a verdict in a fair and impartial manner (i.e. non-biased.^1^) Defendants are entitled to a fair trial and any influence from bias undermines the jury process. Despite this, a whole range of biases exist that influence jurors when they reach verdicts.^4,5^ Bias here has been defined as a factor that produces a preference towards a certain outcome (acquittal or a conviction).^2^ The ramifications of biased and unfair decision-making by jurors can result in injustice. For example, in the case of Sajid Qureshi, he was incarcerated for four years by a jury, where several jurors acknowledged that they had made up their minds before hearing the evidence,^6^ thus highlighting that bias can influence the judgments of jurors.

There are a whole range of opportunities where bias can be introduced into jurors’ decisions. Previous research has highlighted that bias may be introduced by many factors, such as: 1) pre- trial beliefs and attitudes; 2) cognitive bias; and, 3) biased interpretations of evidence by expert witnesses.^7^ Through said factors, bias may play a role in deliberations and may (or may not) impact the decision outcome.

One reason for having a multiple-person jury is that individual biases are intended to be “averaged out”.^11^ However, for this to reasonably occur, a few conditions and assumptions would need to be met. For example, several biases would need to be equally spread out across the jury. If a bias that black people are more likely to commit crime is present in the jury, then there would also need to be a bias that white people are more likely to commit crime, or at least, that black people are no more likely to commit a crime in the jury. These many assumptions and conditions are unlikely. Indeed, proxy research (there is very limited access to actual jury deliberation) on jury decision making suggests the intended ‘averaging’ does not occur.^12^ It should be mentioned here that research on jury deliberations is limited due to a number of factors, including, but not limited to, cost, time constraints, that it is difficult to organize 12 participants to be in same location at once and that researchers do not have access to real world trials or deliberations.^13,14^

The purpose of the current review, therefore, is to highlight and organize the potential sources of bias in juror decision making. To do this, research focusing on three main stages of a trial will be evaluated: 1) pre-evidence presentation (pre-trial publicity); 2) during evidence presentation (cognitive bias and bias from experts) and 3) post-evidence presentation (i.e. during deliberations). The paper will then make recommendations that can reduce the potential for bias and suggest further research into the various ways to combat bias.

Before introducing the literature, it is important to note, that the focus of the current review is on the jury systems in England and Wales, Northern Ireland and Scotland (although Scottish specific issues such as the not proven verdict, will not be discussed here). Despite this focus, some of the research may have been conducted in other countries, such as the United States of America. Therefore, only universal factors that are likely to influence jurors in the jurisdictions mentioned above (pre-trial bias) will be discussed.

## Pre-evidence presentation

### Pre-trial bias

Pre-trial biases and attitudes are among a number of individual differences that influence the verdicts reached by jurors. For example, in addition to bias, personality and political persuasion, can impact juror’s decisions.^2,9,15,16^ The current review will focus on bias and attitudes, however, as the vast majority of the literature has focused on the effects of more global attitudes and how they bias jurors rather than specific political persuasions and/or personality traits.

One early attempt of measuring the influence that pre-trial biases have on verdict choice comes from the Juror Bias Scale (JBS).^17^ The JBS is a 17-item questionnaire that consists of two constructs: 1) the probability of commission; and, 2) reasonable doubt. The probability of commission construct measures prior beliefs and attitudes surrounding evidence. It reflects the extent to which an individual believes that accused people committed the crimes they are accused of. It highlights how guilty the juror may perceive the defendant to be . Nine items were found to adequately measure the probability of commission construct. An example of question relating to the construct include: “defense lawyers don't really care about guilt or innocence, they are just in business to make money”.^18^ Again, the probability of commission construct taps into attitudes relating to a conviction bias, where jurors believe that accused individuals are likely to be guilty, with higher scores indicating a bias towards the prosecution.

The reasonable doubt construct, which is constructed through eight- items, measures how certain the juror needs to be before convicting.^17^ For example, one item in this construct is: “For serious crimes like murder, a defendant should be found guilty so long as there is a 90% chance that he committed the crime”.^18^ Scores on the JBS (from both constructs) can vary from 17 to 85, with high scores indicating a prosecution bias, and low scores highlighting a defence bias. Lecci and Myers^9^ found that the scale accounted for 11.6% of the variance in pre-deliberation verdicts and 6.1% of the variance in post-deliberation verdicts; for more information on analysis, please see Lecci and Myers.^9^ This means that the degree of bias shown by the juror has a notable influence on the verdict they give.

Since the juror bias scale was developed, further research has measured the effects of a number of pre-trial biases and their impact on the decision making of jurors. Lecci and Myers^9,19^ developed the Pre-Trial Juror Attitude Questionnaire (PJAQ), which is made up of six separate constructs or biases: 1) conviction proneness; 2) system confidence; 3) cynicism towards the defence; 4) social justice; 5) racial bias; 6) innate criminality (p. 623). Lecci and Myers^9^ found through the PJAQ that pre-trial biases can be used to predict verdict tendencies. For instance, the PJAQ predicted 21% and 15.1% of the variance in verdict choice at both the pre-deliberation and post-deliberation stage respectively.

Although, all types of bias are not equal, with some of the above constructs having stronger relationships with juror outcomes when compared to others.^9^ For example, conviction proneness (*r* = .39) had a stronger relationship with the pre-deliberation verdicts than other constructs such as system confidence (*r* = .34), social justice (*r* = .15) and innate criminality (*r* = .16)^9^ Interesting, some constructs, such as cynicism towards the defence (*r* = .12) and racial bias (*r* = .06), had no significant association with pre-deliberation outcomes; the role of the latter may have been attenuated due to race not being salient in the mock crime.^9^ Therefore, certain biases may play more of a role in juror decision making than others.

Recent research has utilised the PJAQ when investigating the effects that pre-trial biases have on juror verdicts. For instance, Lundrigan et al.^2^ aimed to investigate if the effect of pre- trial biases on verdict tendencies were mediated by juror interpretations of ‘*beyond reasonable doubt*’. First they found that the PJAQ, on its own, could explain 18.2% of the variance in verdicts that were given by individual jurors, and that by combining the PJAQ with the JBS and the Revised Legal Attitudes Questionnaire (RLAQ-23; a measure of legal authoritarianism) 21.2% of the verdict variance could be explained. Second, it was found that interpretations of ‘*beyond reasonable doubt’* significantly predicted the verdicts ultimately given by jurors. Third, it was found that the PJAQ was a significant predictor of ‘*beyond reasonable doubt’* interpretations, with jurors who are more prone to conviction than acquittal having a lower standard of proof required before they are ready to convict. When ‘*beyond reasonable doubt’* interpretations were controlled for, the predictive ability of the PJAQ over verdict tendencies decreased, but remained significant. These finding suggest that juror interpretations of ‘*beyond reasonable doubt*’ may act as a partial mediator between pre-trial biases and attitudes and the verdict that is ultimately given.

Bias can also enter the courtroom from the presence of pre-trial publicity. Pre-trial publicity was once a type of biasing information that would only influence high profile cases and celebrity trials. However, in the current digital age, jurors could be infected with biasing information regarding any defendant through information that is shared on the internet or on social media sites; a type of viral bias.^20^ Further, Pre-trial publicity has consistently been shown to bias juror judgements in mock trials.^9,19^

In summary, pre-trial biases appear to effect verdict outcome in jury decision making. Although some of the effects of pre-trial bias may be small, bias has a tendency to snowball as it interacts with other elements of a decision.^8^

## During evidence presentation

### Cognitive bias

Cognitive bias is a hypernym that can be utilised to describe the subjective perceptions of individuals which may influence the decisions they make and how they interact with the world.^21^ Further, cognitive bias is produced by: 1) Homo Sapiens having a limited cognitive capacity and thus striving for efficiency when making decisions; and 2) personal and subjectively perceived experiences gained from the environment.^24^ Our cognitive capacity is spared through the use of cognitive short-cuts, such as heuristics. Although mostly useful, heuristics, and their associated biases, can sometimes lead to errors in judgements.^25^ The phenomenon of cognitive bias has been studied in a plethora of different applied environments (e.g. forensic, legal, medicine, and financial) and it has been shown that laypersons, as well as experts, are not immune to the effects of cognitive bias.^8,24,28^

In a landmark study by Carlson and Russo,^7^ the effects that cognitive bias (specifically pre-decisional distortion) has on jurors was investigated. Pre-decisional distortion was defined as: *“jurors’ biased interpretation of new evidence to support whichever verdict is tentatively favored as a trial progresses.”* (P.91). Carlson and Russo^7^ found in two separate studies (the first with a student sample, and the second with a sample of individuals who were selected for jury duty) that jurors tended to favour a verdict before all the evidence was presented. This pre-decisional preference towards a verdict caused jurors to distort how they interpreted the subsequent evidence that was presented, meaning that they perceived the evidence to favour the verdict that was currently leading.

Pre-decisional distortion and “confirmation” bias can also be triggered by the sorts of pre-trial biases and pre- trial information, discussed earlier. For instance, jurors in a negative pre-trial publicity condition (in regards to the defendant), in comparison to control participants, had a preference towards the prosecution (making the guilty verdict their leading verdict).^32^ This preference caused them to distort the evidence to support the prosecution. Jurors in the negative pre-trial publicity condition were also statistically more likely to give guilty verdicts than jurors in the control condition. Similarly, De La Fuente, De La Fuente, and García^33^ found that pre-trial bias influenced jurors when evidence was ambiguous but not when the evidence favoured the prosecution. This is because when evidence is ambiguous it is easier for jurors to distort their interpretation of the evidence to favour their own preferences. Jury trials are inherently ambiguous, as police investigations with strong evidence are likely to lead to confessions of guilt and police investigations with weak evidence against a suspect are unlikely to reach the courtroom.^28^ Further, the adversarial system leads to trials being ambiguous,^34^ with two opposing sides competing to convince jurors that their version of events is closer to the truth than the other side’s. The ambiguity of jury trials creates the perfect environment for cognitive bias to thrive.

The elaboration likelihood model^35^ may explain why bias may play a larger role when the evidence is ambiguous. In this model, its suggests that when the environment leads to a high elaboration likelihood (that is when the environment is not cognitively taxing), individuals are much more likely to be motivated to use cognitive resources in order to engage in the debate and attend to relevant information, thus allowing them to make a decision based on the evidence.^35^ However, when the environment (through little prior knowledge relating to the decision domain or a lack of interest regarding the consequences) leads to a low elaboration likelihood, decision makers are much more likely to reserve cognition through superficially processing the information or relying on prior attitudes or beliefs (i.e. potential biases) when making the decision.^35^ Therefore, in ambiguous environments, jurors, who are not legal experts, may be overwhelmed by the legal terminology being directed at them and confused due to adversarial system of the courtroom. This cognitively taxing environment may cause jurors to have a low elaboration likelihood and thus utilise bias when reaching their verdict.

Cognitive processes and pre-trial attitudes also interact with defendant and victim characteristics to further amplify the effects that bias has in the courtroom. For instance, the representativeness heuristic causes decision makers to equate the similarity between a description and a possible outcome with the likelihood of that particular outcome being correct.^27^ In other words, the more the decision description is able to explain an outcome, the higher the likelihood by which the decision makers judge an outcome to be the correct one, which then leads decision makers to ignore base rate statistics and be subjected to the representativeness bias (i.e. stereotyping.^27^) Therefore, the frugal cue usage of the representativeness heuristic, combined with previous knowledge (e.g. pre-trial bias or attitude) regarding stereotypical actions of out-groups can lead to biased and erroneous decision making. That can then lead to injustices when applied to juror decision making.^36,37^

Previous research has also shown that the interaction between cognitive bias and defendant/victim characteristics can have a negative impact on juror decision making outcomes. For example, ethnic minority groups that are commonly associated in the media with crime are also more likely to be given guilty verdicts in mock juror studies.^38^ Indeed, in a meta-analysis on racial bias on verdict decisions in mock jury studies, Mitchell et al.^38^ examined 34 studies with 7397 participants. A small but reliable racial bias effect was identified, which was stronger if there was a continuous scale rather than a dichotomous choice (e.g. guilty/not-guilty) and if there were no judicial instructions provided. Therefore, pre-trial biases, such as racial biases, are likely to influence how jurors’ stereotype various ethnic minorities, such biases will then influence how novel information is interpreted, and consequently, the final verdict that is given by jurors.

### Bias originating from expert witnesses

Expert testimony is the process by which an expert assists a jury in understanding the evidence presented to them. Jurors must evaluate the testimony of experts in order to make a decision.^39^ Criminal cases often involve multiple experts on both side of the adversarial system.^40^ Experts are selected differently in various legal systems and jurisdictions, but are generally required to evidence their relevant expertise and be able to communicate that expertise in a courtroom situation. They are typically a member of a relevant regulatory body.^41^

Expert testimony has a major influence on juror perception of evidence strength, but this influence is impacted on by many additional factors. These include but are not limited to: the type and complexity of evidence they are giving testimony on (e.g. eyewitness, footwear, DNA;^42^) the type of expertise they are communicating (e.g. clinical or actuarial;^43^) the characteristics of the expert (e.g. gender, appearance, attractiveness;^39^) the conditions of their testimony (e.g. pay rate, frequency of testimony;^44^) their manner of presenting (e.g. categorical like high/low, or likelihood ratios and numerical expressions;^45^) and their willingness/ability to testify about their own doubts and biases.^46^ In this section, when discussing expert testimony, we will be specifically relating to forensic scientists, but this section is likely to generalise to other areas of expert testimony.

The influence that expert witnesses have on jurors is not always positive (i.e. lead to fairer and more accurate decisions), as expert witnesses are unlikely to be rational and impartial decision makers themselves. Previous research has highlighted that task-irrelevant contextual information influences judgements across a number of forensic domains (e.g. fingerprint examination and DNA mixture interpretation.^29^) The effects of biasing information on forensic science judgments has traditionally been seen as negative,^47^ but may lead to accurate decisions in particular scenarios.^48^ Nevertheless, the utilisation of task- irrelevant contextual information by forensic scientists does cause issues at the jury level, and can lead to a snowballing effect of bias in the criminal justice system, thus creating a paradox in logic.^49^ For instance, in accordance with Bayesian norms, jurors should integrate separate pieces of information (e.g. a confession and DNA evidence) independently of one another.^49^ If jurors correctly do this, and the forensic scientist’s evaluation of the forensic evidence was aided by their knowledge of a confession, the jurors think they are integrating each piece of information separately, when in reality each piece of evidence is related; this is known as the criminalist paradox.^49^ Consequently, biased perceptions of evidence by forensic scientists may lead to non-logical judgements being made by the jury.

Despite the subjectivity of forensic evidence interpretation, the mere presence of forensic evidence, such as DNA, in the court room is likely to influence the juror. Further, the presentation of DNA evidence makes cases much more likely to reach court and much more likely for a conviction to occur.^50^ DNA popularity may be based on its marketing as a mechanism that produces either a correct result or no result.^51^ Furthermore, the subjectivity of expert witnesses when they are evaluating forensic evidence, combined with the perceived strength of forensic science by the public, is likely to lead to the effects of bias snowballing from the expert witness to the jury, which may deny a defendant the right to a fair trial. This possibility is exacerbated by the difficulty in presenting forensic evidence in a manner which does not further influence jury decision making.^52^

Another avenue where bias can enter from expert witnesses relates to instruction bias. Expert witnesses are commonly employed by either the prosecution or defence, and work closely with the side that employs them.^53^ It has been suggested that this may make expert witnesses non-neutral and influence the testimony they give, which may then snowball and influence jurors. Further, Murrie, Boccaccini, Guarnera, and Rufino^54^ found that when forensic psychologist and psychiatrists were deceived into thinking they were consulting for the defence or the prosecution that their risk assessment scores significantly differed, with higher scores being given to consultants for the prosecution when compared to consultants for the defence. This problem is not unique to expert witnesses and/or forensic scientists, as police officers and eyewitnesses are as equally likely to impact jurors with their own biased interpretations of events.^55^

### Post-evidence presentation

Legal scholars commonly cite jury deliberations as an important factor that helps to attenuate the effects of bias in the criminal justice system.^34,56^ The logic behind this argument is that by randomly selecting a group (usually 12; but 15 in Scotland^57^) of jurors from the general public, a number of jurors with various biases and beliefs will be selected and that these biases will cancel each other out.^11,44^ It has also been suggested that deliberations allow jurors to focus more on the “facts” of the case rather than on assumptions and will allow more extreme positions to be scrutinised by the collective.^44^

Some research does support the claim that jury deliberations help to attenuate the effects of bias. For instance, Taylor^58^ found in a mock murder trial that negative pre-trial publicity influenced individual juror judgements, with more guilty verdicts being given in the negative pre-trial publicity condition in comparison to the other three conditions (no pre-trial publicity, neutral pre-trial publicity and positive pre-trial publicity). However, the biasing effects of pre- trial publicity were reduced by jurors participating a jury deliberation, with a similar number of guilty verdicts being given across all four pre-trial publicity conditions.^58^ Taylor^58^ also found that the deliberation process attenuated the effects that biasing information had on jurors. Therefore, there is some evidence to suggest that deliberations may help the courts to mitigate bias in jurors.^58^

However, there are two major research areas that have produced evidence that counters the claim that juries (or groups) are more rational (i.e. less biased) than individuals: 1) classical psychological research on group decision making; and 2) jury decision making research. Each of these points will be addressed in turn. First, classical psychological research (e.g. groupthink and group polarization) highlights that group decision making can lead to poor decision performance and extreme, and/or biased, positions (relative to each of the individuals) being taken.^59,60^

Second, some previous juror research has highlighted that deliberations do not reduce the biasing effects that pre-trial publicity has on juror outcomes. For instance, a study by Ruva and Guenther^61^ highlighted that jury deliberations may not attenuate the pre-trial biases that originate because of negative pre-trial publicity. Their study had two aims: 1) investigating the effects of group deliberations on bias (i.e. does it lead to a leniency bias); 2) do juries reach more extreme positions when compared to jurors (i.e. group polarisation). They found that jurors who were not exposed to the negative pre-trial publicity were more likely to favour acquittal verdicts post-deliberations (i.e. participate in a leniency bias). However, jurors who deliberated and had been exposed to the negative pre-trial publicity were worse at source monitoring (e.g. jurors were more likely to misattribute negative pre-trial publicity as forming part of the trial information). Deliberations were also shown not to influence measures of guilt when negative pre-trial publicity had been presented to jurors.^61^ Consequently, group deliberations were found to introduce source monitoring errors and did not decrease the effects of biasing information.

However, it is much more likely that some individual jurors are more rational than a jury and vice versa. For example, we could assume that through randomly sampling jurors that a normal distribution of biases are recruited into a jury. We could also assume that the jury decision is an aggregate of the beliefs of a collective of jurors (which is an assumption as some jurors may have more influence than other.^16^) If we made both assumptions, we would expect 50% of jurors on average to be more rational than juries and 50% of jurors on average to be less rational than juries. Therefore, juries are more rational than some jurors, but some jurors are subsequently more rational than juries.

The composition of biases with juries also seems to be an important factor to consider when evaluating the effects that deliberations have on the usage of biases in juror judgements. For instance, in a study by De La Fuente et al.,^33^ deliberations increased the differences between juries made up of pro-defence jurors when compared to juries made up of pro-prosecution jurors. In their study, mock jurors completed the JBS and were allocated to either pro-defence or pro-prosecution juries based on their answers to the questionnaire. The results of the study highlighted that jurors with a pro-prosecution (pro-defence) bias gave significantly more (fewer) guilty verdicts post deliberation in comparison to pre-deliberation when the evidence presented was ambiguous. Therefore, juries that are homogeneous in relation to pre- trial biases are problematic and may lead to the effects of bias being amplified within the courtroom.^33^ Further, jury selection procedures that allow juries to be made up of a group of heterogeneous jurors may aid the criminal justice system in tackling bias; this topic will be addressed further in the recommendations section.

From a limited amount of access to deliberation rooms and a great deal of proxy research it can be suggested that the intended ‘averaging’ out of biases does not always occur.^11^ More research that incorporates group deliberations into their designs is consequently needed for the academic community to get a fuller and more nuanced understanding of the effects that group deliberations may have on bias.

### Recommendations

In relation to pre-trial bias, one method of attenuating bias would be for courtrooms to utilise jury selection (or Voir Dire) procedures based upon scientific instruments such as the PJAQ.^2,9,33^ The PJAQ, and similar inventories that measure bias, would allow courtrooms to screen out jurors with extreme views or perceptions,^62^ which may be particularly important in relation to rape trials. By filtering jurors with a preference for a particular verdict (guilty or not guilty) out of a jury, there would be less of a tendency for pre- trial biases to guide evidence evaluation in trials, thus limiting the effects that these biases may have on the final verdict choices of jurors.^2,9,33^ However, caution must be taken in any filtration and selection of jurors, as this can be easily misused (intentionally or not).

Obviously, there are practical issues in regards to implanting such a strategy. Some biases may not influence certain trial types, for example an individual who beliefs in rape myths may still be perfectly capable of serving as a juror in homicide trial. The decision to remove certain jurors will depend on the complex interplay between the characteristics of the crime, the defendant, the evidence presented and the juror (alongside the attitudes and experiences they bring). More research is needed in this area to establish how pre-trial biases interact with the factors above in order to inform how tools such as PJAQ can be used for jury selection. Another issue is, whose role would it be to make decisions regarding jury selection? Would it be legal professionals? This may not attenuate bias to the courtroom but instead could just add a new avenue for bias to enter trials. Further, these individuals may not have the expertise and are already stretched due to workload demands. We instead suggest that psychologists, who have expertise in using tools such as PJAQ be employed to select jurors. However, before said changes were made, it would be advisable to conduct research to assess the effectiveness of such a change and to consult legal professionals on their perceptions to said recommendation.

Cognitive bias, however, has proven more difficult to deal with. This is because cognitive bias is produced by natural cognitive structures (such as schemas and heuristics) and are consequently an artefact of thinking and reasoning.^25,27^ Therefore, it is impossible to remove cognitive bias entirely from the juror decision process. The effects of cognitive bias have been shown to be attenuated by the presentation of strong evidence.^30,34^ Nevertheless, as previously mentioned, trials in the adversarial system are inherently ambiguous,^28^ leaving the potential for bias to *always* have an impact on juror decisions.^62^

One way of tackling cognitive bias, however, may be to improve the quality of the evidence presented to jurors from expert witnesses. Expert testimony could be improved through a number of different strategies: 1) increased training to help experts communicate their testimony in a clear and logical way that legal laypersons understand; 2) using independent experts that are not associated with either side of the adversarial process;^8^ 3) improving codes of conduct for expert witnesses^63^ Again, mock jury research should be conducted to assess the effectiveness of these approaches in reducing the effects of bias in the courtroom.

More research is needed which tackles the effects that jury deliberations have on bias. Nevertheless, the stratified sampling of jurors through instruments, such as PJAQ, may also help to decrease the chances that juries with extreme homogenous biases (i.e. juries consisting of pro-prosecution or pro-defence jurors) will be selected.^35^ Meaning that juries could be composed, through a selection procedure, of heterogeneous jurors. This may allow jury deliberations to be more fit for purpose and act as method of attenuating the effects of bias in jurors (as jurors will be critiquing, rather than confirming, each other’s beliefs). Also, how jurors deliberate can be structured in a way that enhances the benefits of group decisions, while minimizing negative group dynamics.

Another method of attenuating the effects of bias in jurors would be to remove biased testimony (surrounding events and evidence) from the courtroom. We propose that the ‘war against bias’ should also be fought where evidence is collected, interpreted, and presented by forensic scientists; as this may reduce the effects of negative biases snowballing throughout the legal system. We suggest three fronts. First, the study of forensic bias needs to investigate the effects of non-relevant contextual information on forensic scientists in ecologically valid settings and utilise commonly used principals of experimental study (e.g. randomisation procedures.^64^) Further, previous studies on contextual bias in forensic examiners has not included control groups, analysed data using inferential statistics, not included information regarding randomisation procedures and/or conducted the research in artificial settings with non-practioners.^64^ Research of higher quality is therefore needed in order to combat the effects of biased decision making in forensic examiners.^64^ Such research will help to establish mechanisms for reducing the effects of bias in the evaluation of forensic evidence. Second, we propose that techniques, such as Linear Sequential Unmasking (LSU), are used by forensic laboratories to minimise the potential for contextual information to have a negative effect on the decision making performances of forensic scientists.^65^

LSU is a procedure “*that requires examiners not only to first examine the trace evidence in isolation from the reference material, but also provides a balanced restriction on the changes that are permitted post exposure to the reference material.*”^65^ (P.3). In other words, in LSU, examiners would examine the evidence at the crime scene (e.g. fingerprints) without knowledge of reference material from the accused (e.g. their fingerprints). During this period the examiner would state unique features of the trace evidence, ensuring that analysis of trace evidence is not contaminated by knowledge of the reference material. Then, examiners can analyse the reference material, any changes to their initial analysis should then be documented to ensure transparency.^65^

LSU has been expanded to also minimise noise,^66^ and improving decision making in general, by sequencing the task-needed information also by its level of objectivity as well as level of relevance.^67^ Other techniques that could be employed are evidence line-ups, where examiners need to match up the trace material with the correct reference material, despite the presence of foil evidence.^68^ However, the effects of bias on forensic examiners is likely to be multifactorial and several methods should be employed to attenuate bias at this stage of the criminal justice system in order to stop the effects of bias snowballing and impacting on juror judgements.

A third recommendation is that psychologists should educate jurors and legal professionals about the impacts of bias and task-irrelevant contextual information on the judgements of forensic scientists.^69^ In relation to legal professionals, this could be conducted as part of their continuous personal development and conducted on an annual basis. For jurors, a short informative video, designed by psychologists, could be shown to jurors prior a trial. However, research should be conducted to test if such a training course would be effective at making jurors and legal professionals more aware about the impacts of bias. Further, training courses and/or informative videos could also educate jurors, and legal professionals, about the potential for information to be linked through the *potentially* biased evaluation of the forensic scientist (i.e. the criminalist paradox.^49,69^) Through providing lawyers with said information it will give them a tool to question the objectivity of forensic scientists and thus may decrease the influence that biased forensic evidence may have on juror judgements. For instance, research has found that when expert witnesses are questioned and cross-examined about the influence of bias and task-irrelevant contextual information on their judgements, jurors perceive the expert witness as less credible and are more likely to acquit.^70^

In regards to the testimony given by police officers and eyewitnesses it is unlikely that bias can be removed from their interpretation of events. For instance, the police may generate a biased perception of a suspect through finding out their fingerprints matched with the prints found on the weapon. This bias may then influence how they investigate a crime and, subsequently, what they tell the jury, which could lead to jurors generating their own biases against the defendant. Eyewitnesses’ perceptions of events may be influenced by factors preceding the criminal event. For example, a fight between two adults (each equally to blame) may be perceived as an assault by one of the adults if the beginning of the altercation is missed by the witness. In a similar vain to the above paragraph, psychologists should educate legal professionals on bias and the effects that contextual information may have on how individuals (eyewitnesses and police) may interpret events and evidence. This may allow jurors to evaluate the impact that bias has had on the witness’s interpretation of events.

Despite our critique here of bias in jurors, we are not necessarily suggesting that judges should exclusively make decisions on guilt. Research has shown that judges: 1) use heuristic decision making strategies; 2) make similar judgements to jurors; and 3) when they make different judgements to jurors, they are more likely to give a guilty verdict, which may increase incidences of injustice.^71^ In addition, judges have been shown to: 4) demonstrate similar rates of implicit bias regarding race as members of the general public;^74^ 5) show socioeconomic biases, most prominently in child custody cases;^75^ and, 6) to hold biases based on religion.^76^ Previous research has also highlighted that expertise may have a paradoxical effect on bias; with experts developing schema, stereotypes and base rates that bias how they perceive the information relevant to the decision.^77^ Consequently, the replacement of jurors with expert judges may not, by itself, attenuate the role that bias plays in the criminal justice system. Despite these findings, a substantial minority of legal professionals indicate a preference for panels of judges to replace juries.^57^

In summary, bias enters the courtroom in a number of ways and the effects that it has on juror decision making is multi-factorial. Due to this a number of strategies, some listed above, could be employed to aid in the fight against bias. However, before recommendations can be implemented, more research is needed to assess the effectiveness of certain bias reducing strategies (both independently and when interacting with other strategies). For this, governments and legal bodies need to take the effects of bias more seriously and fund high quality jury studies aimed at tacking the effects of bias in the criminal justice system.

## Conclusion

In conclusion, bias is a multifaced phenomenon that can be introduced to the process of juror making through a number of avenues. Previous research has highlighted that jurors may be biased by pre-trial attitudes and beliefs and cognitive processes. Further, biases may also be introduced into the courtroom through witness (both expert and non-expert) testimony that is biased. Each element discussed in this review has the potential to interact with each other and consequentially cause the effects of bias to snowball throughout the legal system. Legal scholars commonly suggest that jury deliberations are a successful method of attenuating bias in jurors. However, there is limited and contradictory evidence here. In this review, four main practical recommendations can be made to attenuate the effects of bias on jurors. First, tools such as PJAQ should be used to ensure that juries consist of a heterogenous group of jurors, each with differing beliefs and biases. Therefore, pre-trial biases in jurors may cancel each other out in the deliberation room. Second, improve the quality of expert testimony through increased training and using independent experts that are not associated with either side of the adversarial process. Third, research into the effects of contextual information on forensic decision making is continued with a greater emphasis on experimental control and ecologically valid settings, and measures such as linear sequential unmasking are utilised by forensic laboratories. Fourth, jurors and legal actors (judges/lawyers) should be provided with some knowledge relating to the effects that bias can have on witness (both expert and not) interpretations of situations and evidence. Only then can the objectivity of witnesses be assessed in the courtroom.
